# Sputnik Planitia as an impactor remnant indicative of an ancient rocky mascon in an oceanless Pluto

**DOI:** 10.1038/s41550-024-02248-1

**Published:** 2024-04-15

**Authors:** Harry A. Ballantyne, Erik Asphaug, C. Adeene Denton, Alexandre Emsenhuber, Martin Jutzi

**Affiliations:** 1 Space Research and Planetary Sciences, Physics Institute, University of Bern, Bern, Switzerland; 2 Lunar and Planetary Laboratory, University of Arizona, Tucson, AZ USA; 3 University Observatory, Ludwig-Maximilians Universität München, Munich, Germany

**Keywords:** Asteroids, comets and Kuiper belt, Geomorphology, Early solar system

## Abstract

Pluto’s surface is dominated by the huge, pear-shaped basin Sputnik Planitia. It appears to be of impact origin, but modelling has not yet explained its peculiar geometry. We propose an impact mechanism that reproduces its topographic shape while also explaining its alignment near the Pluto–Charon axis. Using three-dimensional hydrodynamic simulations to model realistic collisions, we provide a hypothesis that does not rely upon a cold, stiff crust atop a contrarily liquid ocean where a differentiated ~730 km ice–rock impactor collides at low-velocity into a subsolidus Pluto-like target. The result is a new geologic region dominated by impactor material, namely a basin that (in a 30° collision) closely reproduces the morphology of Sputnik Planitia, and a captured rocky impactor core that has penetrated the ice to accrete as a substantial, strength-supported mascon. This provides an alternative explanation for Sputnik Planitia’s equatorial alignment and illustrates a regime in which strength effects, in low-velocity collisions between trans-Neptunian objects, lead to impactor-dominated regions on the surface and at depth.

## Abstract (web summary)

Foreign material delivered as a giant impact can dominate large portions of icy dwarf planets, according to impact simulations. This scenario may explain the peculiar shape and location of the Sputnik Planitia region on Pluto, without the need for a present-day subsurface ocean.

## Main

In 2015, the New Horizons space probe revealed Pluto’s surface to be geologically complex^1^. It is dominated by an ~1,200 km × 2,000 km, nitrogen-ice-filled basin named Sputnik Planitia (SP)^1^ whose central plains are 3–4 km below its surroundings^2^. These values correspond to the surface of the nitrogen ice, whose thickness is unknown. The quasi-elliptical shape and mountainous rim resemble a degraded impact basin^3^, which has motivated several studies into its formation using computational impact models^4,5^. These suggest that a 400-km-diameter impactor could produce an ~800 km basin comparable in diameter to SP; however, they are two-dimensional simulations of head-on collisions and cannot reproduce the feature’s elongated morphology.

Here we use three-dimensional impact simulations to consider more realistic impact angles and impactor structures and the complex material and dynamical interactions that ensue. We use the smoothed-particle hydrocode SPHLATCH (ref. ^6^), which is the basis for a wide range of planetary collisional studies^7–10^, and apply equations of state (EOSs) for rock (dunite) and water ice to represent the bodies. Strength has been shown to be important, even up to Mars-size targets^10,11^, so we include shear strength and plasticity in all our simulations using a Drucker–Prager-like yield criterion. Yield strength is a function of pressure and temperature, so is pronounced in cold materials and, thus, applicable to Pluto. The specific strength parameters follow those of previous impact studies^12–15^ and are given in Extended Data Table 1. Further geophysical parameters, such as cohesion, tensile fracture and porosity, are important in collisions at the scale of asteroids and comets^16^ but are negligible in giant basin formation and are not included here.

Pluto is represented as a differentiated body composed of a rocky core and a water-ice mantle. The former constitutes two thirds of the total mass in agreement with observations by New Horizons^17^. The physical state of the H_2_O mantle is uncertain, with recent studies advocating for subsurface water that lies below a thick ice shell^18,19^. One particular motivation for this scenario is the location of SP. The formation of a large topographic depression is expected to induce true polar wander, reorienting Pluto to position the depression closer to the nearest pole. Instead, SP is near Pluto’s equator and opposite its large, tidally locked, satellite Charon, implying the presence of a mass concentration (‘mascon’)^20^ rather than a deficit. The prevailing hypothesis is that a subsurface ocean can explain such a mascon, as the excavation of SP caused an uplift of denser salty water from below^18,19^. The crater structure and subsurface ocean must persist into the present, because if the ocean had solidified or the ice crust had relaxed viscously, then the mascon would have vanished. Preventing the ocean from freezing necessitates factors such as an exceptionally high ammonia concentration to lower the melting temperature of ice^18^ or an ever-present layer of gas hydrates that insulates the warm liquid ocean against the gelid ice shell^19,21^.

**Extended Data Fig. 1:**
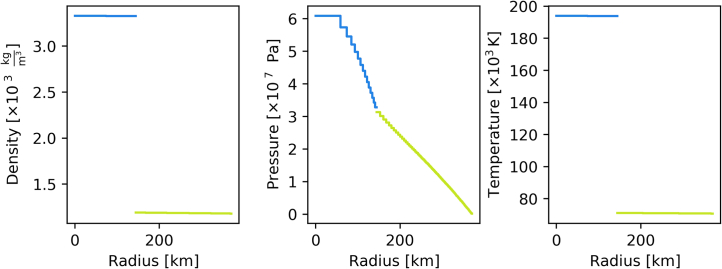
Interior profiles for the nominal impactor. (15% core mass fraction). Colour signifies composition, where blue represents rock material and green represents ice.

**Extended Data Fig. 2:**
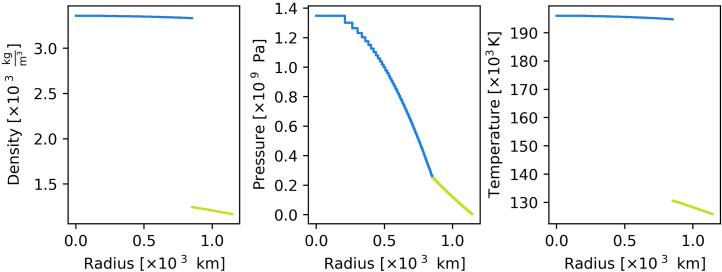
Interior profiles for the nominal target body. Colour signifies composition, where blue represents rock material and green represents ice.

**Extended Data Fig. 3:**
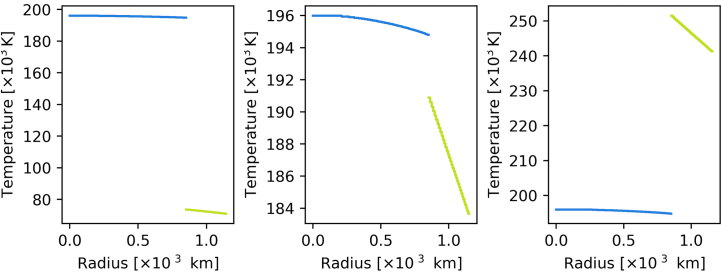
Alternative temperature profiles investigated for Pluto’s mantle. Alternative temperature profiles investigated (all but the nominal case) for Pluto’s mantle. Colour signifies composition, where blue represents rock material and green represents ice. Note that only the internal energy (and thus temperature) properties of the ice-mantle are different to the nominal case (Extended Data Figure 2); any differences in the rock-core properties, or in the density or pressure of the ice mantle, are negligible.

To bracket the possible pre-impact conditions, our suite of simulations includes Pluto targets with solid rock cores (dunite EOS) and solid ice mantles between 70 and 250 K and cases with subsurface oceans with temperature profiles consistent with a water EOS. Impactors are modelled using the same materials, but their parameters are less constrained. Kuiper belt objects exhibit a wide range of diameters^22–25^, and despite observations of over 2,000 of these objects^26^, meaningful mass estimates are available for only a handful^27^, making their densities and compositions quite uncertain. For this work, we consider solid impactors between 400 and 1,100 km in diameter, with compositions ranging from rock to ice and to in-between (5–66 wt% rock surrounded by ice). In the latter, multi-material cases, we model the impactor as fully differentiated, as is the case for Pluto. In reality, however, the degree of differentiation decreases with size^28^. Partial differentiation has even been suggested for bodies as large as Charon^29–32^. The implications of this discrepancy are considered in the ʻDiscussionʼ. In any case, the smaller size of the impactors implies that there is a cooler temperature than that of Pluto, and they are, therefore, modelled with ice-mantle temperatures of ~70 K. The precise interior profiles used for the nominal impactor and target are given in Extended Data Figs. 1 and 2, respectively. Other target temperature profiles explored are given in Extended Data Fig. 3.

We considered impacts with angles ranging from 0° to 45° and velocities of 1.0*v*_es_ to 1.4*v*_esc_. Here, *v*_esc_ represents the mutual escape speed for the two bodies (~1.2 km s^−1^), and is given by ${v}_{{{{\rm{esc}}}}}=\sqrt{\frac{2G({m}_{{{{\rm{imp}}}}}+{m}_{{{{\rm{tar}}}}})}{{r}_{{{{\rm{imp}}}}}+{r}_{{{{\rm{tar}}}}}}}$, where *G* denotes the gravitational constant, *m* is mass and *r* is radius, and the subscripts imp and tar signify the impactor and target, respectively. The impacts are slower than the speed of sound in geologic ice, ~2–4 km s^−1^, so shocks play a minor role. This velocity range is like that of Pluto–Charon formation scenarios^33^. If SP formed when Pluto was much closer to the Sun^26^, random encounters would probably have been faster; in this case our scenarios might apply to a collision with a massive satellite of Pluto or to the last major event in Pluto’s original accretion, because the mass ratios, angles and velocities lie within the ‘merging’ impact regime^34,35^.

The main impact sequence (shock wave traversal and dissipation and transient crater formation and collapse) occurs quickly, within the first ~2 h after impact. The vast majority of displaced material settles soon thereafter, with only a small amount of ejecta on target-crossing orbits that will re-impact at later times. Our simulations were extended for 6 h after impact, such that deformation has ceased. Any remaining lofted ejecta composes less than 0.5% of the total mass.

## Results

**Fig. 1:**
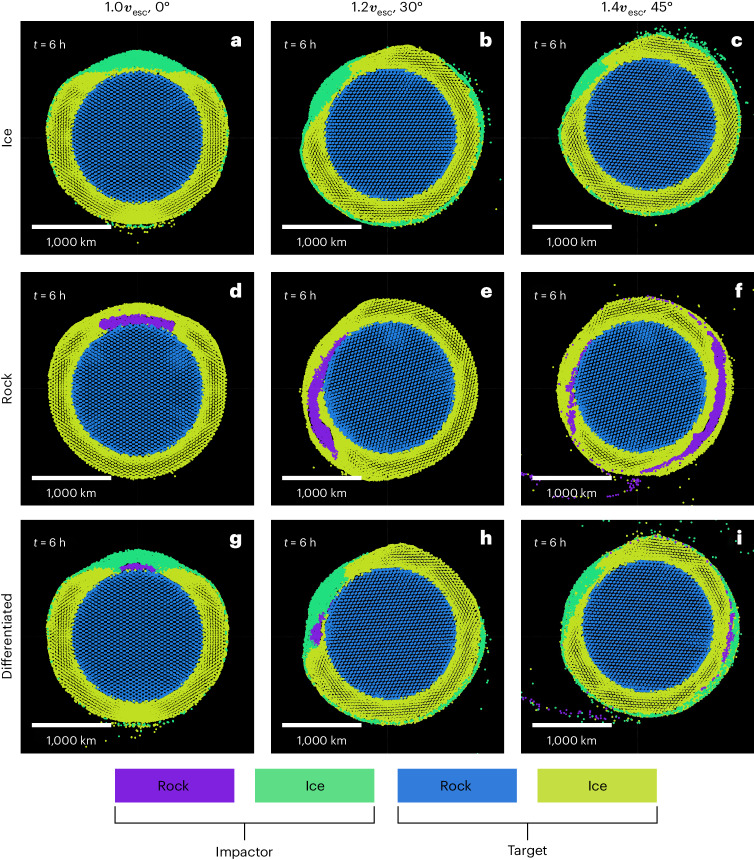
Results of various impact simulations based on smoothed-particle hydrodynamics that highlight important regimes in the explored parameter space. **a**–**i**, Each plot shows the resulting body *t* = 6 h after initial contact as a cross-section of the impact plane with a thickness of 300 km. Colour denotes the composition and source parent body of the material, as labelled. The impactor masses are the same in each case, so that the ice impactor is the largest, approximately 730 km in diameter. The bodies are initially solid, and their temperature profiles correspond to the nominal case. The differentiated impactors have a core mass fraction of 15%. For an illustration of the initial set-up of these simulations, see Supplementary Fig. 1. **a**, Ice impactor, 1.0*v*_esc_, 0°. **b**, Ice impactor, 1.2*v*_esc_, 30°. **c**, Ice impactor, 1.4*v*_esc_, 45°. **d**, Rock impactor, 1.0*v*_esc_, 0°. **e**, Rock impactor, 1.2*v*_esc_, 30°. **f**, Rock impactor, 1.4*v*_esc_, 45°. **g**, Differentiated impactor, 1.0*v*_esc_, 0°. **h**, Differentiated impactor, 1.2*v*_esc_, 30°. **i**, Differentiated impactor, 1.4*v*_esc_, 45°.

Upon examination of the impact outcomes, distinct trends become evident. Figure 1 shows cross sections of the target for a variety of cases, from head-on at impact velocity 1.0*v*_esc_ (left) to oblique at impact velocity 1.4*v*_esc_ (right), for impactors of ice, rock and a differentiated ice–rock composition (top to bottom). In each case, a transient crater forms in Pluto’s mantle (larger for the larger-diameter ice impactor), which by *t* = 6 h after initial contact has collapsed. The ice impactor (Fig. 1a–c) experiences profound deformation upon colliding with Pluto’s ice mantle, spreading out to become a ‘splat’ akin to impactor distributions proposed to explain the Moon’s striking nearside–farside dichotomy^36^. The impacting material starts off less compressed and thus somewhat less dense, so remains mostly atop Pluto’s ice throughout the crater collapse. The rock impactor (Fig. 1d–f) is denser, stronger and more difficult to melt and is much more resistant to deformation and breaking up, so instead, it pierces Pluto’s ice mantle, remaining largely intact until it impacts Pluto’s rocky core. Then it spreads out into a hidden, deeply buried splat, a rocky mass atop the core–mantle boundary.

Increasing the impact angle and velocity contribute a downrange motion, so that the distribution of the impactor material becomes asymmetric. In these relatively low-velocity (subsonic) collisions, most of the material of the ice impactor remains above the ice mantle of Pluto regardless of impact angle. For a larger impact angle and velocity, the break-up of the impactor and its continuing momentum can lead to a fraction of it going into orbit and forming a depositional ring along the impact plane where it re-impacts.

Rock impactors also continue downrange in oblique collisions, but being denser, they plough through the icy mantle in a complex interaction. The details of these interactions depend on the material parameters of the colliding bodies and on the physics of mixing of dissimilar materials, with the added complexity that material remains cold and rigid well beyond the transient cavity. Generally speaking, for impact angles ≲30°, a rocky impactor comes to a halt downrange, and if it overcomes the strength of cold ice, it sinks towards the core (for example, Fig. 1e). For oblique collisions (≳30°) and for higher velocities, a rocky impactor can slide and bounce downrange until it comes back out of the icy mantle before breaking up into a ring of impactor material that extends downrange from the impact site (for example, Fig. 1f).

A differentiated impactor (Fig. 1g–i) results in scenarios that effectively combine the features of the ice and rock end members. As the ice is the first material to make contact with Pluto, it shields the impactor core from substantial deformation and heating, so it remains more intact than an impactor without an ice mantle. In the head-on case (Fig. 1g), the rocky core ends up at the core–mantle boundary, whereas the mantle ice fills the transient cavity. A notable distinction from the pure-ice scenario is the considerable amount of impactor ice pulled down to the core–mantle boundary by the impactor core. Consequently, the impact site comprises predominantly impactor material extending down to Pluto’s core.

**Fig. 2:**
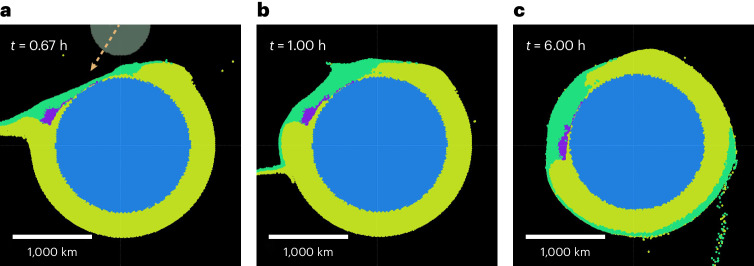
Time series of a simulation based on smoothed-particle hydrodynamics for an ~730-km-diameter impactor, 15% core mass fraction, *v*_coll_ = 1.2*v*_esc_ and an impact angle of 30°. **a**–**c**, Slices through the impact plane. Colours indicate material composition and source parent body, with purple and green indicating impactor rock and ice, and blue and yellow indicating target rock and ice, respectively, as in Fig. 1. The faded body shows the size and position of the impactor at the moment of impact, with the arrow indicating the impact velocity. **a**, Shortly after impact, the transient crater is still present. The impactor core has impacted Pluto’s core and continues downrange. **b**, The transient SP crater has collapsed and been infilled with ice from the impactor. Beneath, the impactor core has nearly come to rest along the ice–rock boundary. **c**, State after impact, *t* = 6 h. The rocky impactor core is at rest under the narrow southern end of SP.

We find that these intermediate parameters, specifically with a core mass fraction of 5–30%, impact angle between 15° and 30° and an impactor diameter of around 700 km (comparable to the mass of asteroid Vesta), yield the most promising conditions for generating SP. An example in this range is shown in Fig. 2, which is our case that best fits the observations. The impactor first excavates the mantle of the impact site (Fig. 2a) to produce an elliptical transient crater. The core, shielded by the ice, stays largely intact as it is deflected through Pluto’s ice shell, ultimately halting while remaining embedded within the mantle (Fig. 2b). The transient crater becomes filled with infalling impactor ice, and the impactor core settles towards Pluto’s core to produce a mascon near the core–mantle boundary (Fig. 2c).

**Fig. 3:**
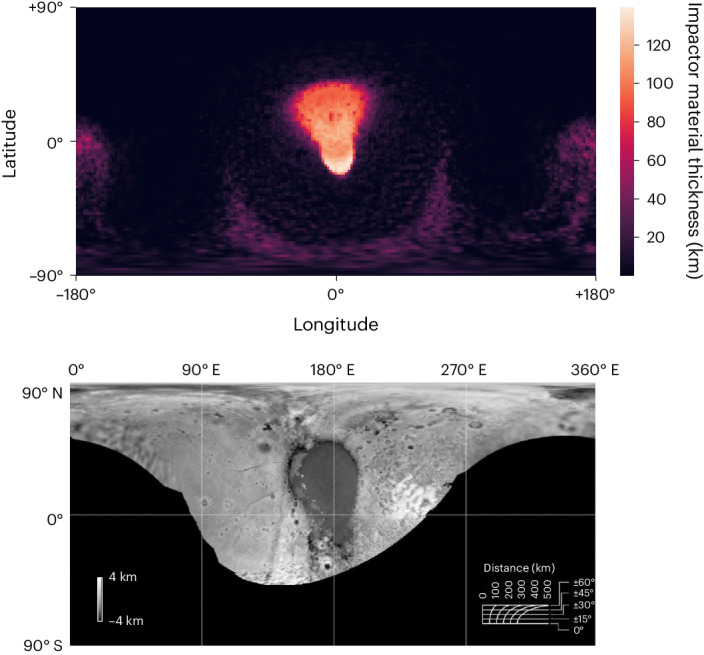
The distribution of impactor material after impact compared to Pluto’s observed elevation distribution. Top, distribution of impactor material after the nominal collision (same as Fig. 2), displayed as an equirectangular projection of thickness to a maximum depth of ~150 km. The spherical reference frame has been chosen to best match the location and orientation of SP today. North of the equator, the distribution is nearly elliptical, corresponding to the impactor’s first point of contact and the subsequent transient crater. South of the equator lies the distribution’s pointed ‘tail’, which is caused by the impactor core sliding through the Plutonian mantle. Bottom, Pluto’s observed elevation distribution. SP is the feature in the centre. For a further comparison, see Extended Data Fig. 4. Figure reproduced with permission from ref. ^2^, Elsevier.

**Extended Data Fig. 4:**
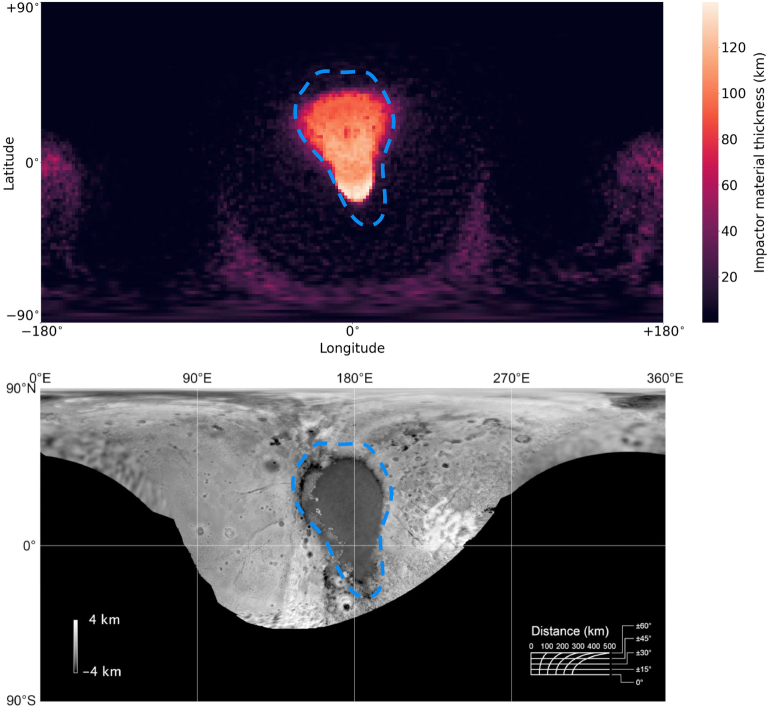
The post-impact distribution of impactor material compared to Pluto’s observed elevation distribution, with a dashed-line outline highlighting the shape of SP. (Top) The distribution of impactor material after the nominal collision (same as Fig. 3), displayed as an equirectangular projection of thickness to a maximum depth of ~ 150 km. (Bottom) Pluto’s observed elevation distribution, where SP is the feature outlined by a blue dashed-line in the centre.The SP outline follows that of ref. ^2^; for a direct comparison of these outlines, see Supplementary Figure 4. Figure reproduced with permission from ref. ^2^, Elsevier.

The final outcome of these cases is a region of Pluto entirely dominated by impactor material with a shape in longitude–latitude space remarkably like that of SP, as shown in Fig. 3 (and Extended Data Fig. 4), which displays the final mass distribution of impactor material and a comparison with SP. The more circular shape in the north corresponds to the point of collision, where the transient cavity opens and collapses. The pointed configuration in the southern region corresponds to the notable downrange movement of the impactor core through the target mantle, until it comes to rest atop the target core.

The precise distribution of the impactor-dominated region is controlled by the impactor’s initial parameters. Its size determines the broad scale of the distribution, whereas its core mass fraction controls the breadth of the ‘tail’ of our teardrop-like distribution, owing to its dependence on core radius. The impact velocity also affects the scale of the transient crater, thus influencing the size of the elliptical region of impactor ice that forms at the immediate point of impact. However, its primary influence is on the length of the downrange tail. Higher velocities cause the core to travel a greater distance through the mantle before reaching a standstill and falling back towards the core–mantle boundary to create downrange disruption and surface deposition.

Considering all these factors, we find that a 15% rock, ~730 km impactor striking Pluto at 30° with an impact velocity, *v*_coll_, of 1.2*v*_esc_ (~6 km s^−1^) produces an impactor-dominated region at the impact site that best matches the planimetric morphology of SP (as in Fig. 3).

In each of these cases, the rocky core of the impactor settles beneath the southernmost, narrow tail region of the impact site and becomes buried beneath the impactor mantle above. We propose that this material serves as the mascon responsible for SP’s current location on Pluto. The substantial mass excess provided by the dense rock is expected to drive SP’s position towards Pluto’s equator through true polar wander, in a similar fashion as described in previous studies^18–20^. In this scenario, we expect that the tail of SP (that is the region with the strongest mass excess) would align close to Pluto’s equator, as is observed today.

**Fig. 4:**
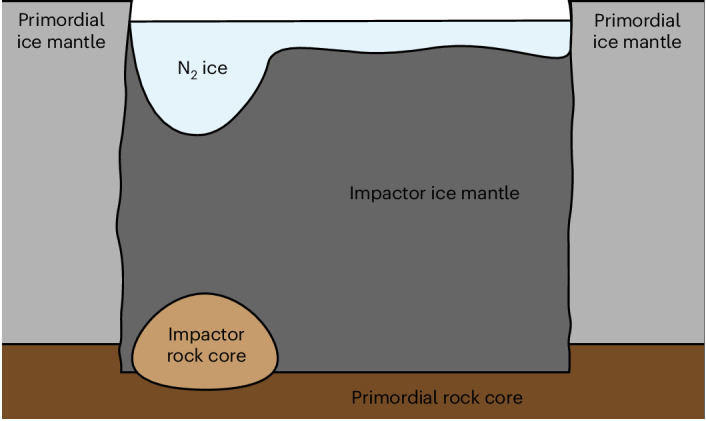
Schematic diagram depicting the proposed geologic structure beneath SP. Colour signifies the composition, as indicated by the labels. The surface of SP corresponds with the surface of the N_2_ ice. The ice mantle directly below SP is of impactor origin, according to the simulations. If the impactor mantle has a somewhat greater density than the ice mantle of Pluto, then the entire SP region will sink towards isostasy to create a regional depression. The impactor’s rock core ends up buried beneath the southernmost region of SP, where it remains. The higher density of this mascon, and the resulting deeper topography and reservoir of N_2_ ice above it, leads to a positive gravity anomaly that establishes and maintains SP’s near-equatorial position through true polar wander.

Finally, the SP-shaped, impactor-dominated region must produce the basin associated with SP today. We propose that this could be due to a contrast in composition between the impactor and Pluto. If the impactor mantle was of a greater density than the Pluto mantle, the impactor-dominated region would apply a greater load on Pluto’s silicate core. This would cause it to sink to lower elevations than its surroundings as it moves towards isostasy, thus leading to the observed depression. As the load on Pluto’s core would be greatest in the region containing the impactor core, this region would initially be the deepest depression within SP; however, N_2_ ice would would rapidly accumulate in the basin^37^, smoothing out the surface and concealing the mantle’s previous topography. A diagram depicting the proposed internal structure of SP can be seen in Fig. 4.

A contrast in density between the impactor and target material might be expected due to the different degrees of differentiation inside each body based on their very different masses. The impactor may, thus, have had a less-pure, more dusty ice mantle than the purer, and thus, lower-density, ice mantle of the pre-impact Pluto. Likewise, a less completely differentiated impactor core might have retained more lower-density constituents and been less dense, with a buoyancy against the higher-density rocky core of Pluto, further aiding its long-term stability at the core–mantle boundary.

## Discussion

These results may explain SP’s unusual morphology and the proposed positive gravity contribution without the need for a subsurface ocean. The existence of an ocean would depend on the temperature profile of Pluto, which is not well constrained. Our nominal target has a solid ice mantle that starts at ~130 K and a solid rock core at ~195 K (Extended Data Fig. 2). This is consistent with ice-shell temperatures in previous SP formation models^4,5^ and with temperature predictions from studies on the thermal evolution of Pluto, at least for early times (within roughly 250–500 Myr post-formation)^38,39^ when impacts of this magnitude were probable^40^. The rock-core temperature is consistent with that from some previous models^39,41^, but it has been suggested that after ~500 Myr it may reach ~500–700 K due to radiogenic heating^39,41,42^. We, therefore, repeated our best-fitting impact simulation using a core temperature of ~500 K but found only negligible differences.

To explain SP, the basin shape and profile as predicted by these models must be supported over geologic time. In cold solid materials, the basin structure (SP itself and the dense buried mascon provided by the impactor’s core) would be supported by strength. Isostatic readjustments would occur around these emplaced materials, with the mascon, thus, underlying the deepest region of the basin, and the impactor mantle (if it is slightly denser than Pluto’s mantle) would underlie a broader depression. N_2_ ice would quickly accumulate inside the basin^37,43^, potentially contributing further to the positive mass anomaly that we propose drove SP into its current position through true polar wander. The southernmost, narrow section of SP, directly above the mascon in our simulations, would be forced closest to the equator, matching observations.

**Extended Data Fig. 5:**
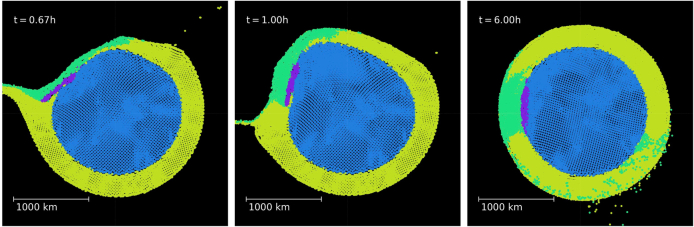
The nominal impact scenario as a time series without including strength. Slice through the impact plane. Colours indicate material composition and source parent body, with purple and green indicating impactor rock and ice, and blue and yellow indicating target rock and ice, respectively, as in Fig. 1. Substantial deformation of Pluto’s core occurs throughout the impact event, in stark contrast to our simulations that incorporate strength.

**Extended Data Fig. 6:**
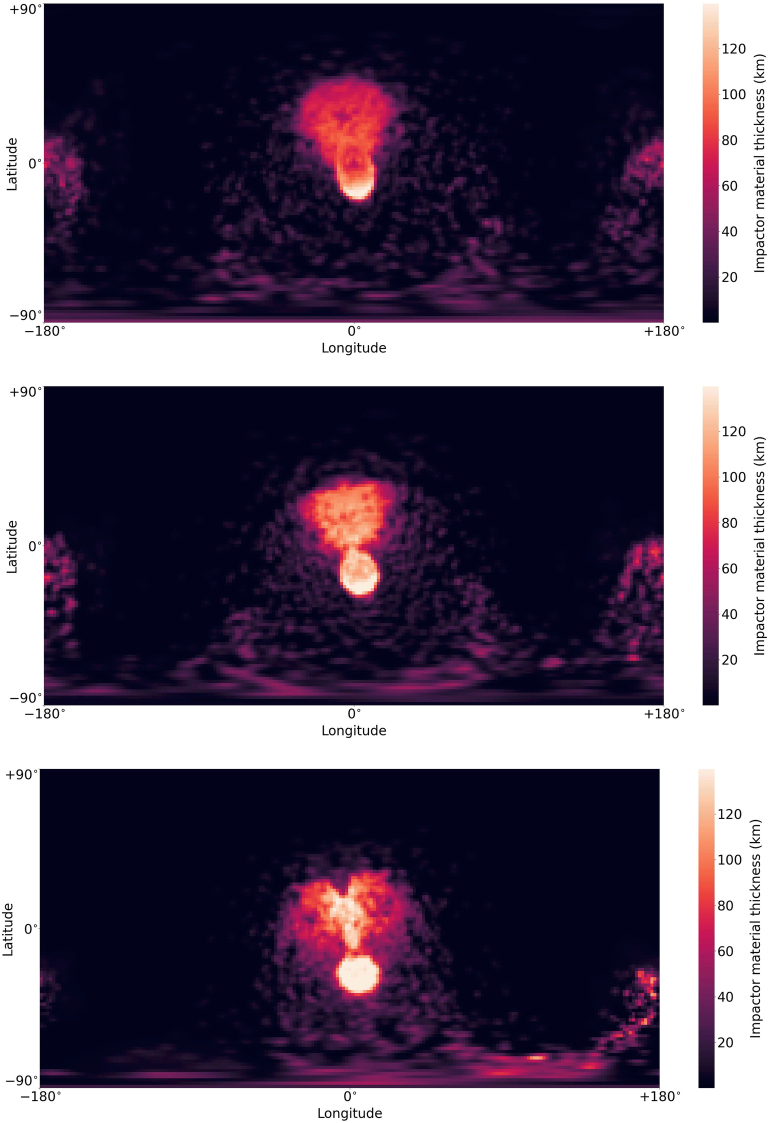
The distribution of impactor material after the nominal collision for all additional temperature profiles explored for a solid Pluto mantle. The distribution is displayed as an equirectangular projection of thickness to a maximum depth of ~ 150 km. Mantle temperature increases from the top plot to the bottom (that is ~ 70 K (top), ~ 190 K (middle), ~ 250 K (bottom), following the profiles in Extended Data Figure 3 from left to right). These cases can also be viewed as an interior cross-section in Supplementary Figure 5.

Temperature can affect our proposed scenario, even for solid targets, because the formation of SP depends on material strength (Extended Data Fig. 5), which is weaker for higher temperature bodies and zero in an oceanic layer. We, therefore, tested the sensitivity of our impact mechanism to these effects by repeating the nominal impact with a broad range of target ice-mantle temperatures. For our coldest targets, which start at ~70 K, the final material distribution of SP (Extended Data Fig. 6, top) looks very like our nominal case (Fig. 3). For somewhat warmer ice, ~190 K (Extended Data Fig. 6, middle), the final distribution also looks similar; however, a stronger uplift of the target mantle occurs beneath the impactor core. For even warmer but still solid ice, ~250 K, the impact induces substantial melting, resulting in much more mixing of Pluto’s original ice and water with that of the impactor. Consequently, the final distribution of impactor material becomes less distinct, having a more elliptical shape (Extended Data Fig. 6, bottom). These results indicate that our proposed impact mechanism is sensitive only to temperatures near (≲50 K below) or above the solidus, at which the material strength is not adequately maintained. At lower temperatures, the impact scenario remains very much like our reference case. Therefore, differences between our simplified temperature profiles (Extended Data Fig. 2) and that of the real Pluto would not affect our results as long as Pluto’s interior was sufficiently subsolidus at the time of impact.

We also considered targets that have a subsurface water ocean 50, 100 or 150 km thick, as per earlier investigations^5^. The ocean thicknesses correspond to a temperature–pressure profile consistent with the water EOS, with a transition to a solid ice shell in the exterior (see Supplementary Fig. 2 for the exact interior profiles used).

**Fig. 5:**
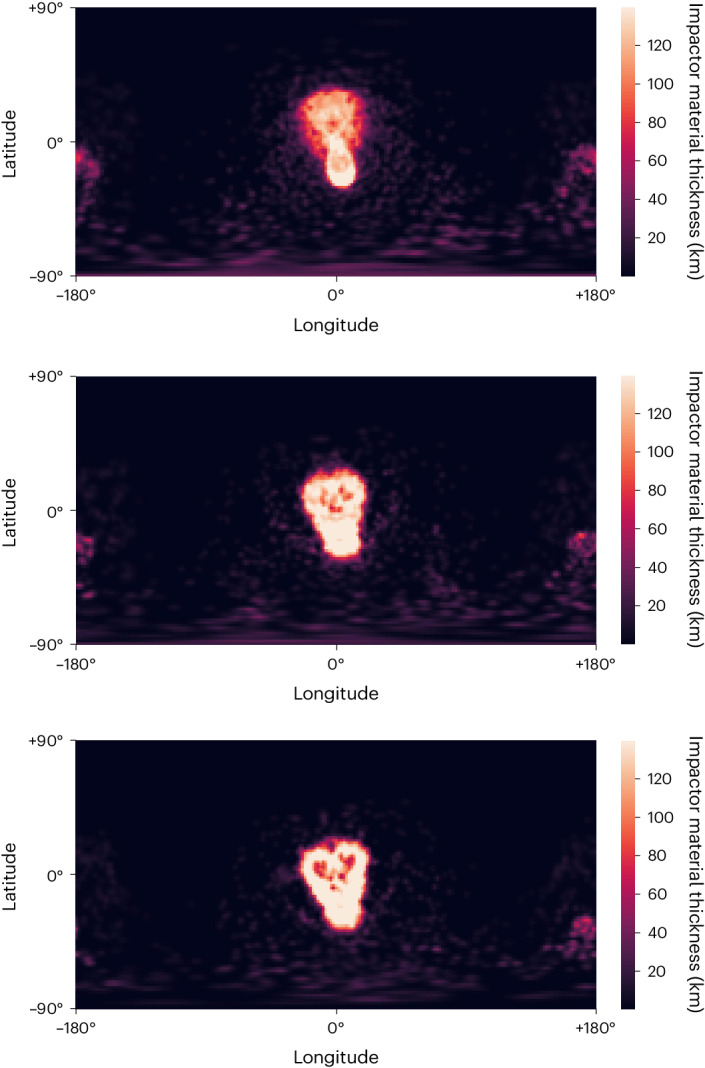
Equirectangular projections of the impactor material distribution after impact for each subsurface ocean thickness. Top, subsurface ocean thickness of 50 km. Middle, subsurface ocean thickness of 100 km. Bottom, subsurface ocean thickness of 150 km. These cases can also be viewed as an interior cross-section in Supplementary Fig. 3.

**Extended Data Fig. 7:**
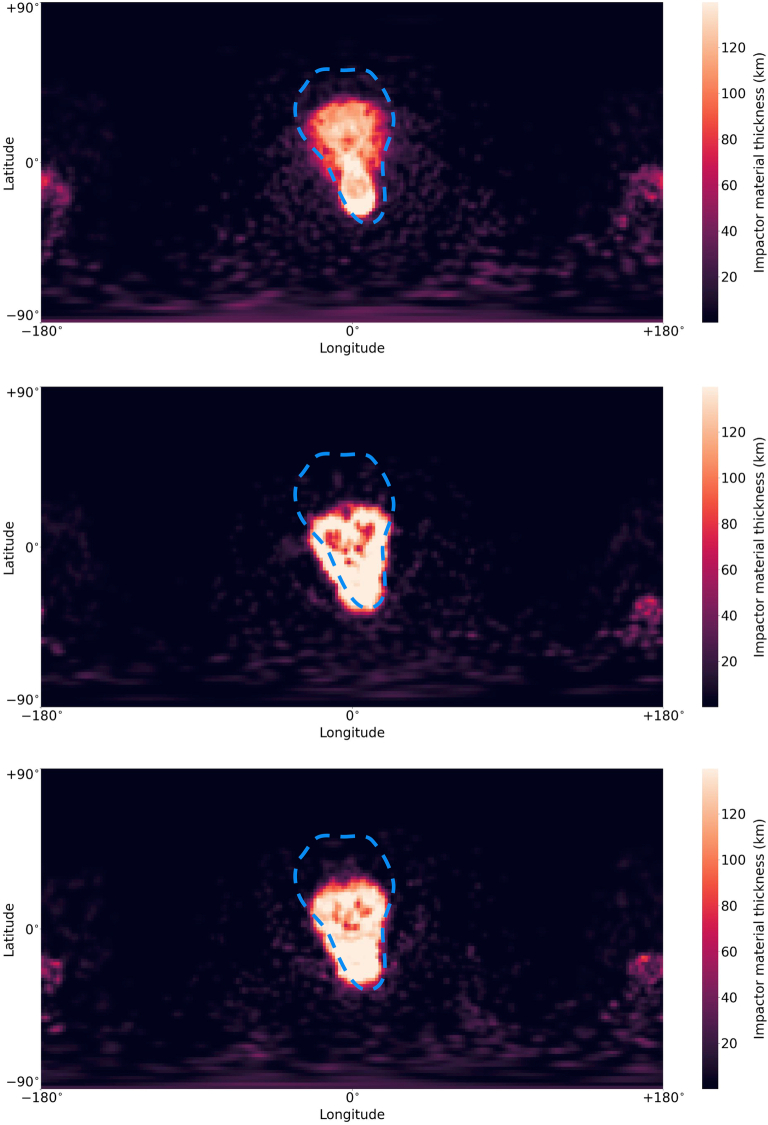
Equirectangular projection of the post-impact impactor material distribution for each subsurface ocean thickness, with a dashed-line outline highlighting the shape of SP. Equirectangular projection of the post-impact impactor material distribution for each subsurface ocean thickness (50 km, 100 km and 150 km from top to bottom), with a dashed-line outline highlighting the shape of SP. The SP outline follows that of ref. ^2^; for a direct comparison of these outlines, see Supplementary Figure 4.

For a starting ocean 50 km thick, we observe that the impactor material distribution still resembles SP quite well, though with a shape that is more compact than for a solid target. The elliptical region produced by the impactor’s contact and compression into Pluto is particularly diminished (Fig. 5). This pattern persists for 100-km-thick and 150-km-thick oceans, which end up with an even smaller contrast between the elliptical area around the region of contact and the tail sculpted by the continuing impactor core. These instances with thicker oceans bear minimal resemblance to SP’s morphology (Extended Data Fig. 7), and we, therefore, deem them unlikely.

Overall, we find that a thin, target ocean (≲50 km thick) is favoured among our candidate scenarios. This aligns with previous subsurface ocean thickness estimates derived from thermal evolution models of Pluto that assumed an ammonia content like that of other Kuiper belt objectss (≲1%) and a typical reference viscosity for ice (10^14^ Pa s)^38,41,44–47^. If the influence of ammonia proves negligible, Pluto might not possess a subsurface ocean at all^41,44^, in accordance with our nominal case.

In our proposed scenario, the impact that produces SP leads to a band of re-impacting ejecta in the impact plane (Fig. 2). Reference ^2^ suggested that re-impacting SP material could be related to the origin of the complex ridge–trough system (RTS) that stretches north to south across Pluto’s surface and is interrupted at SP. SP and RTS have an ~10° offset in longitude–latitude space, but this could possibly be accounted for by pre-impact rotation (see ref. ^48^ for a similar scenario involving impact ejecta on Vesta).

RTS has also been proposed to be a paleoequator^2^, meaning it could be linked to the true polar wander caused by SP (ref. ^49^). In this case, the impact plane of SP would be very close to the paleoequatorial plane, suggesting that the SP-forming impact may have contributed considerably to Pluto’s rotation. Clearly, future work including both pre-impact rotation and subsequent true polar wander calculations are necessary to further understand SP’s origin and its implications for other features such as RTS.

Other follow-up work should include modelling the gravity field associated with the positive anomaly of the buried impactor core and exploring other possible compositions for the impactor and target. These results could then inform a sophisticated geophysical model to investigate the long-term evolution of SP over geological timescales. This would be particularly important for understanding the proposed isostatic readjustment.

Further impact studies with a wider range of impacts and a more systematic approach should also be performed to better constrain the parameter space of this regime. Higher velocity and more oblique impacts will cause the impactor core to burst through the target planet’s surface and re-impact further downrange than the reference impactor (as in Fig. 1i); however, the limiting speed and angle for this scenario will change for different impactor sizes and compositions.

Finally, we highlight that the impact mechanism outlined in this study is not limited to the formation of SP. Impacts between mostly solid bodies with masses up to that of Pluto and occurring at close to the escape velocity have been shown to generate heterogeneous interior structures that may persist to the current era. The proper interpretation of those structures may, thus, be crucial in discerning the properties (both interior and exterior) of the icy worlds that populate the cold outer fringes of planetary systems.

## Methods

### Smoothed-particle hydrodynamics

The impact simulations were conducted with the three-dimensional smoothed-particle hydrocode SPHLATCH (ref. ^6^). This method represents a continuum by a set of smoothed particles, each representing a fixed mass distributed across a sphere with radius 2*h*, where *h* is the smoothing length. The physical properties (for example density and internal energy) of an individual particle were computed using a kernel-based interpolation method that considers all neighbouring particles within the 2*h*-radius sphere. To ensure that there was a roughly constant neighbour count inside the smoothing sphere of each particle, *h* was variable, with regions of lower density having larger smoothing lengths. Self-gravity was included using a Barnes and Hut tree algorithm^50^. Distant particle contributions were calculated with the multipole approximation (up to the quadrupole moments). For an in-depth description of SPHLATCH, we direct interested readers to ref. ^51^.

A total of 200,000 particles were employed in each simulation. To test for convergence, the best case was performed again using 1,000,000 particles. This high-resolution run was utilized for the best case figures (Figs. 2 and 3); however, only negligible differences were observed between the two resolutions, and thus, we deem all our simulations adequately resolved. Each collision was simulated for 6 h after impact.

### Material properties

Using the software ANEOS (refs. ^52,53^), an EOS was used to calculate pressure and temperature, with input parameters for dunite^12^ and ice^7,54–56^. The latent heat of ice was accounted for within the ANEOS (refs. ^57,58^). The latent heat was not used for dunite, but this did not affect our results as all rocky material remained well below melting temperatures throughout the impact simulations. The material strength was represented by a Drucker–Prager friction yield criterion, which varies as a function of pressure and temperature^16,59^. The strength parameters are consistent with those from previous impact modelling studies^12–15^, as inferred by experiments conducted within the temperature and pressure range relevant to this work. A comprehensive description of the strength model can be found in ref. ^11^ using the corrections of ref. ^10^.

Extended Data Table 1 lists the input parameters used in our strength model. Due to the large, planetary scale of the impacts considered in this study, we considered all material to be fully damaged. Dunite input values are from ref. ^12^. Our ice values are very close to those of ref. ^15^, which were determined by fitting to experimental results for quasi-static shear strength, friction and dynamic tensile strength. Other studies have used substantially higher yield parameters for ice^5,60,61^. Reference ^15^ adjusted the parameters from ref. ^14^ to better reflect the frigid temperatures of large icy satellites like Ganymede and Europa, which suit the conditions considered in this work. We make one modification, namely to the yield strength (*Y*) of damaged (fractured) ice, with the equation of ref. ^62^:

1 $${Y}_\mathrm{d}=\min ({Y}_\mathrm{d,l},{Y}_\mathrm{d,h})$$

with

2 $${Y}_\mathrm{d,l}={\mu}_\mathrm{d,l}P$$

and

3 $${Y}_\mathrm{d,h}={\mu}_\mathrm{d,h}P+{Y}_{0,\mathrm{h}},$$

where *Y*_d,h_ and *Y*_d,l_ represent the yield strength of damaged ice at high and low pressures, respectively, *P* denotes pressure, *μ*_d,h_ and *μ*_d,l_ represent the friction coefficients for damaged ice at high and low pressures, respectively, and *Y*_0,*h*_ represents the *y* intercept for an empirical fit to the experimental results at high pressure.

### Impactor distribution maps

To calculate the regional distribution of impactor material on the final Pluto target, we employed a spherical grid akin to that of ref. ^10^. An impactor mass fraction *β*_imp_ was assigned for each particle. Impactor particles have *β*_imp_ = 1, and the remaining particles that constitute the target have *β*_imp_ = 0. This value can be calculated for any point in space with a smoothed-particle hydrodynamics sum:

4 $${\beta}_{{{{\rm{imp}}}}}({{{\mathbf{x}}}})=\sum_{b}{\beta}_{{{{\rm{imp}}}}}W({{{\mathbf{x}}}}-{{{{\mathbf{x}}}}}_{b},{h}_{b}),$$

where **x** denotes a given spatial point, *b* signifies a particular neighbouring particle and *W* represents the smoothing kernel, which is the cubic B-spline function. The spherical grid was then constructed with a resolution of 7 km in radius and $\frac{\pi }{100}$ in latitude and longitude. *β*_imp_ was calculated for every grid point with equation (4). By summing all *β*_imp_ values at each latitude and longitude coordinate down to a specified depth and multiplying this sum by the grid resolution in radius Δ*r*, the total thickness of impactor material *z*_imp_ was calculated at each coordinate:

5 $${z}_{{{{\rm{imp}}}},\;jk}=\Delta r\mathop{\sum}\limits_{i={i}_{\min }}{\beta }_{{{{\rm{imp}}}},ijk},$$

with subscripts *i*, *j* and *k* denoting the grid cell index for each radius, latitude and longitude, respectively, and ${i}_{\min }$ denoting the index for the specified depth, which we take as ~150 km.

To change the reference frame of our spherical coordinate system, we rotated the particle coordinates before constructing the spherical grid. This method was used on our best-fitting simulation case to match the location and orientation observed of SP, allowing for better visual comparison in Fig. 3. The reference frames of all subsequent impactor distribution maps (Fig. 5 and Extended Data Fig. 6) were also rotated by the same degree.

### Supplementary information

Supplementary Information Supplementary Figs. 1–5.

## Figures and Tables

**Extended Data Table 1:**
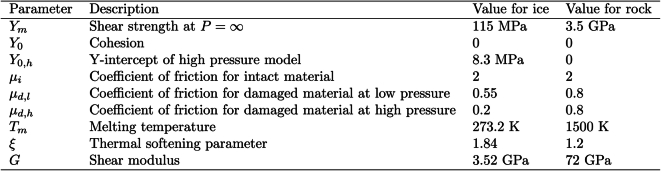
Parameters used in the material strength model

The parameters for rock (dunite) are from ref. ^12^, and those for ice from ref. ^15^ and ref. ^62^. Some more recent studies^5,60,61^ have adopted substantially stronger ice models in the studied regime; however, little justification is provided for those values, and some appear to be erroneous (for example a damaged coefficient of friction, *μ*_*d*_, of 0.8, which should be 0.2 according to their ref. ^62^).

## Data Availability

The simulation data used to create all figures presented in this study has been published on Zenodo, 10.5281/zenodo.10696642 (ref. ^63^).

## Code availability

A compiled version of the SPHLATCH code, along with the necessary input files, can be obtained from the corresponding author upon reasonable request.
