# Supplementary material for: Sputnik Planitia as an impactor remnant indicative of an ancient rocky mascon in an oceanless Pluto
Source: Nat Astron. 2024 Apr 15;8(6):748–55. doi: 10.1038/s41550-024-02248-1 (PMC11189809; doi:10.1038/s41550-024-02248-1)
Supplement: Supplementary file 1 — Supplementary Figs. 1–5. [file 41550_2024_2248_MOESM1_ESM.pdf]

nature astronomy

Article

https://doi.org/10.1038/s41550-024-02248-1

Sputnik Planitia as an impactor remnant indicative of an ancient rocky mascon in an oceanless Pluto

In the format provided by the authors and unedited

Contents

1 **Impact Regime Simulations at Initial Contact** **2**

2 **Additional Subsurface Ocean Figures** **3**

3 **SP Outline Comparison with Schenk et al. (2018)** **4**

4 **Post-Impact Interiors for Different Mantle Temperatures** **5**

1 Impact Regime Simulations at Initial Contact

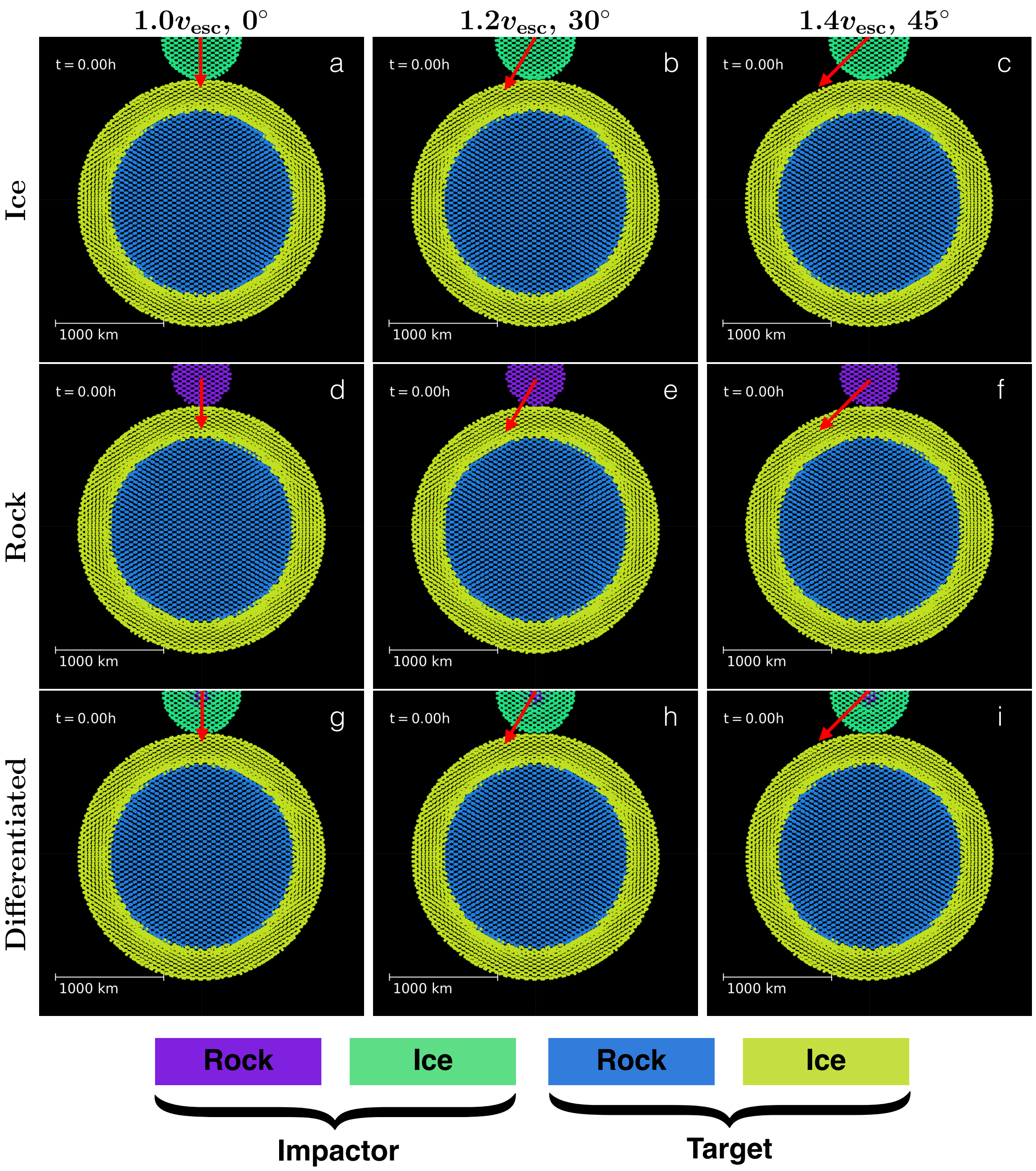

Supplementary Figure 1: Various SPH impact simulations that highlight important regimes in the explored parameter space at $t = 0$, the time of initial contact. Each plot is a slice of thickness 300 km in the impact plane. Colour signifies material composition and parent body, as labelled. The impactor mass is held equal, thus the ice impactor is largest, approximately 730-km diameter. The differentiated impactors have a core mass fraction of 15%. Red arrows indicate the direction and speed of the impact. The layout of the plots and the simulations they represent match those of Fig. 1

2 Additional Subsurface Ocean Figures

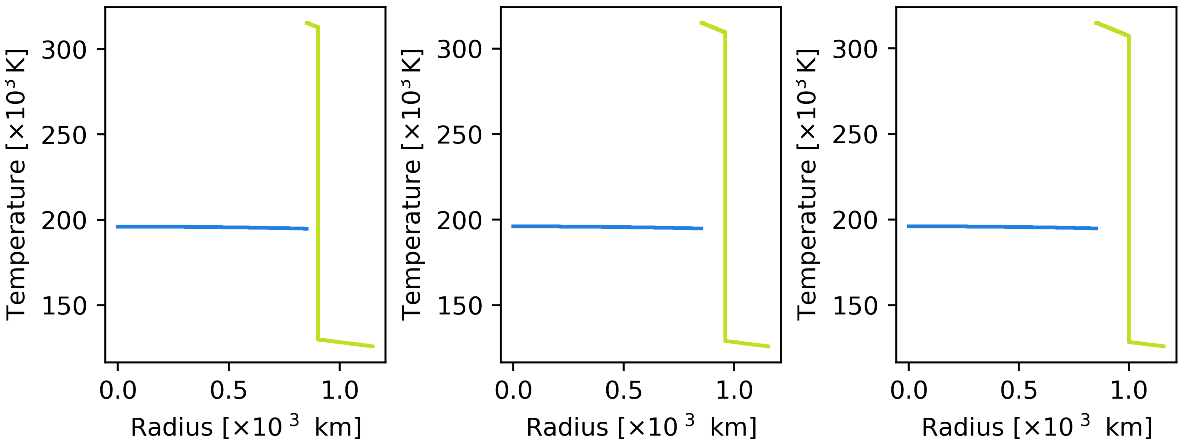

Supplementary Figure 2: Temperature profiles for each subsurface ocean thickness investigated for Pluto (50 km, 100 km and 150 km from left to right). Colour denotes composition, where blue represents rock material and green represents ice.

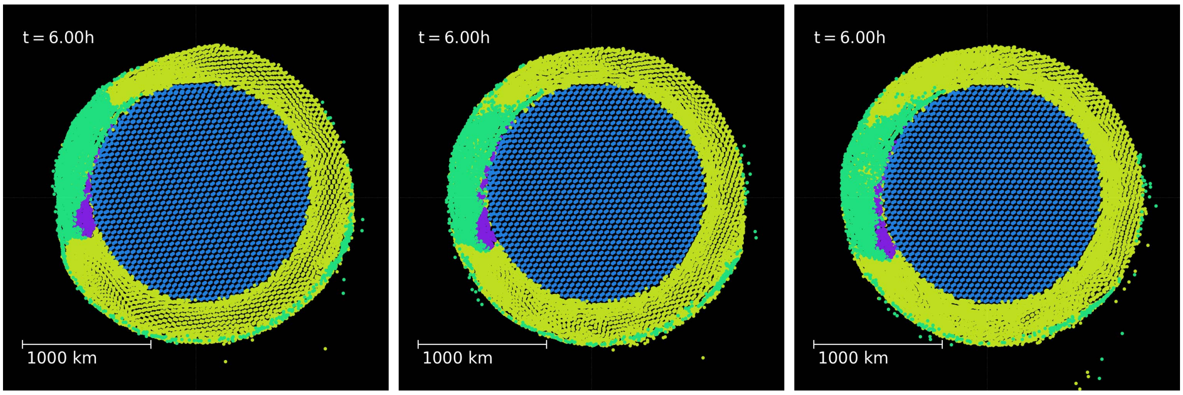

Supplementary Figure 3: 300 km slices in the impact plane showing the distribution of material after the nominal impact for each pre-impact subsurface ocean thickness (50 km, 100 km, 150 km from left to right). Colours indicate material composition and source parent body, with purple and green indicating impactor rock and ice, and blue and yellow indicating target rock and ice, respectively, as in Supplementary Figure 1.

3 SP Outline Comparison with Schenk et al. (2018)

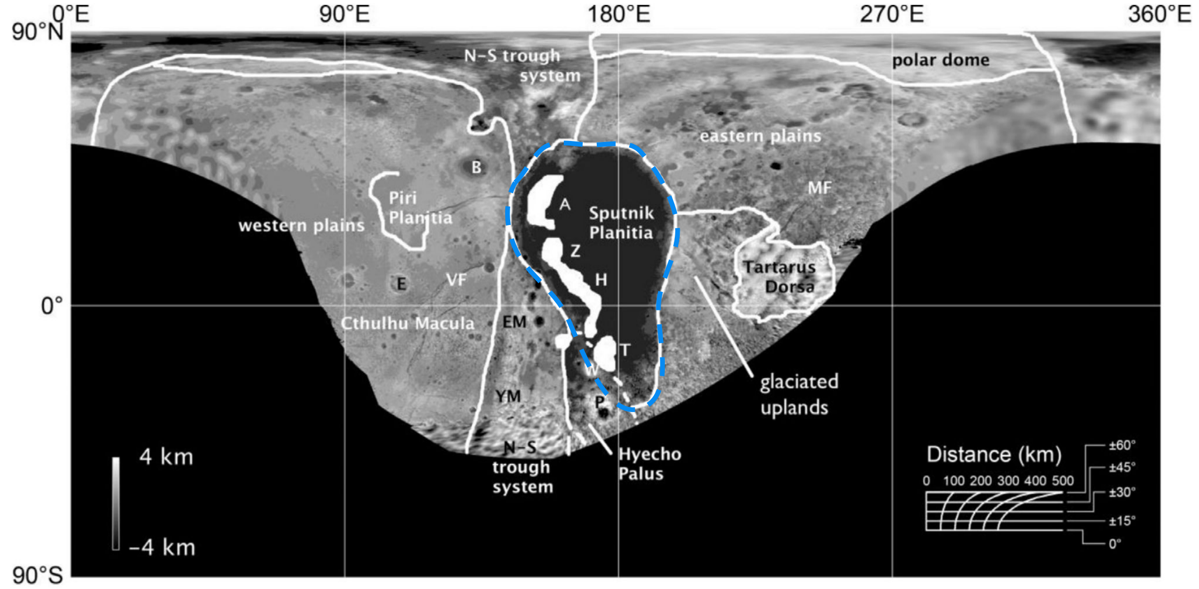

Supplementary Figure 4: Figure 9 of Schenk et al. (2018) outlining the major provinces of Pluto, with the addition of a blue dashed line following the outline of SP used for comparison to the results of this study. Reprinted from Schenk et al. (2018), with permission from Elsevier.

4 Post-Impact Interiors for Different Mantle Temperatures

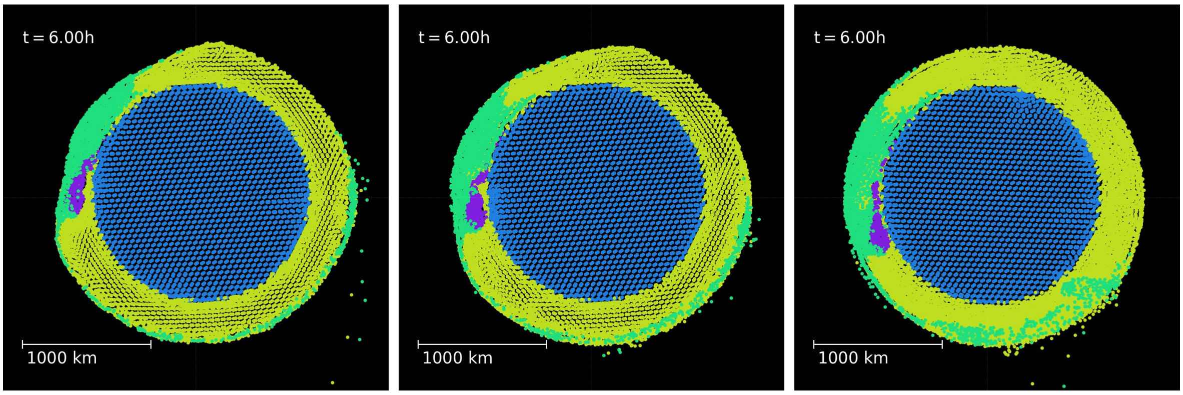

Supplementary Figure 5: 300 km slices in the impact plane showing the distribution of material after the nominal impact for each non-nominal ice mantle temperature. Mantle temperature increases from the top plot to the bottom (i.e. ∼70 K (top), ∼190 K (middle), ∼250 K (bottom), following the profiles in Extended Data Figure 3 from left to right). Colours indicate material composition and source parent body, with purple and green indicating impactor rock and ice, and blue and yellow indicating target rock and ice, respectively, as in Supplementary Figure 1.

References

Schenk, P. M., Beyer, R. A., McKinnon, W. B., Moore, J. M., Spencer, J. R., White, O. L., Singer, K., Nimmo, F., Thomason, C., Lauer, T. R., Robbins, S., Umurhan, O. M., Grundy, W. M., Stern, S. A., Weaver, H. A., Young, L. A., Smith, K. E. & Olkin, C. (2018), ‘Basins, fractures and volcanoes: Global cartography and topography of Pluto from New Horizons’, *Icarus* **314**, 400–433.
